# GNV2-SLAM: vision SLAM system for cowshed inspection robots

**DOI:** 10.3389/frobt.2025.1648309

**Published:** 2025-09-19

**Authors:** Xinwu Du, Tingting Li, Xin Jin, Xiufang Yu, Xiaolin Xie, Chenglin Zhang

**Affiliations:** 1 Longmen Laboratory, Luoyang, China; 2 College of Agricultural Equipment Engineering, Henan University of Science and Technology, Luoyang, China; 3 Collaborative Innovation Center of Machinery Equipment Advanced Manufacturing of Henan Province, Luoyang, China

**Keywords:** SLAM, YOLOv8, GNV2-SLAM, cowshed inspection, computer vision

## Abstract

Simultaneous Localization and Mapping (SLAM) has emerged as one of the foundational technologies enabling mobile robots to achieve autonomous navigation, garnering significant attention in recent years. To address the limitations inherent in traditional SLAM systems when operating within dynamic environments, this paper proposes a new SLAM system named GNV2-SLAM based on ORB-SLAM2, offering an innovative solution for the scenario of cowshed inspection. This innovative system incorporates a lightweight object detection network called GNV2 based on YOLOv8. Additionally, it employs GhostNetv2 as backbone network. The CBAM attention mechanism and SCDown downsampling module were introduced to reduce the model complexity while ensuring detection accuracy. Experimental results indicate that the GNV2 network achieves excellent model compression effects while maintaining high performance: mAP@0.5 increased by 1.04%, reaching a total of 95.19%; model parameters were decreased by 41.95%, computational cost reduced by 36.71%, and the model size shrunk by 40.44%. Moreover, the GNV2-SLAM system incorporates point and line feature extraction techniques, effectively mitigate issues reduced feature point extraction caused by excessive dynamic targets or blurred images. Testing on the TUM dataset demonstrate that GNV2-SLAM significantly outperforms the traditional ORB-SLAM2 system in terms of positioning accuracy and robustness within dynamic environments. Specifically, there was a remarkable reduction of 96.13% in root mean square error (RMSE) for absolute trajectory error (ATE), alongside decreases of 88.36% and 86.19% for translation and rotation drift in relative pose error (RPE), respectively. In terms of tracking evaluation, GNV2-SLAM successfully completes the tracking processing of a single frame image within 30 ms, demonstrating expressive real-time performance and competitiveness. Following the deployment of this system on inspection robots and subsequent experimental trials conducted in the cowshed environment, the results indicate that when the robot operates at speeds of 0.4 m/s and 0.6 m/s, the pose trajectory output by GNV2-SLAM is more consistent with the robot's actual movement trajectory. This study systematically validated the system's significant advantages in target recognition and positioning accuracy through experimental verification, thereby providing a new technical solution for the comprehensive automation of cattle barn inspection tasks.

## 1 Introduction

In autonomous navigation systems of mobile robots, simultaneous localization and mapping (SLAM) concept is widely acknowledged as a core technology that enables robots to achieve accurate self-location and environmental mapping without prior knowledge of the environment (Jia et al., 2022). Particularly, Visual SLAM (VSLAM) method has received special attention due to its low hardware cost and its ability to capture rich environmental details (Islam R. et al., 2023). However, traditional VSLAM methods are generally based on the premise of environment statics (Sun et al., 2022), which faces many challenges in actual environments. Especially in dynamic environments containing moving targets, the problem of extracting erroneous feature information from dynamic objects in turn triggers degradation of localization accuracy or tracking interruptions (Wan Aasim et al., 2022). If there are multiple moving targets or camera motion blur in the environment, traditional VSLAM methods will reduce the number of extracted features, leading to a decrease in system stability and robustness. Taking the inspection environment of cattle sheds in animal husbandry as an example, inspection robots are often disturbed by the movement of cattle or workers when performing inspection tasks. Meanwhile, the cowshed is a structural environment with a large number of line features. In order to ensure the reliable operation of the SLAM system in such complex scenarios, it is necessary to design a SLAM system that can identify and eliminate dynamic feature points in real-time, and combine with line features in the environment to improve overall positioning accuracy and robustness.

Deep Learning-based VSLAM is regarded as a promising solution to address the challenges of dynamic environments (Song et al., 2022). It can recognize predefined dynamic target categories, and providing the system with their semantic labels and coordinate information. By proposing characteristics on dynamic targets, it improves the localization accuracy in dynamic environments, which lays the foundation for autonomous detection of inspection robots.

Red Green Blue-Depth (RGB-D) camera can accurately obtain depth information through sensor measurements, while their color images can be used for visual tasks such as target recognition and image segmentation. Although the image segmentation technique is effective in eliminating the interference of dynamic objects to the SLAM system, its high computational overhead tends to affect the real-time performance of the system (Liu and Miura, 2021). Therefore, YOLO (You Only Look Once), as a single-stage efficient object detection framework, has gradually become the preferred solution in dynamic environment SLAM systems. The structurally optimized YOLO model can provide localization accuracy close to that of image segmentation methods while maintaining a high detection speed, thus striking a good balance between accuracy and real-time performance (Zhang et al., 2022). When there are multiple moving objects on the image or when there is image blurring, the number of extracted point and line features is reduced. Whereas, in the absence of texture or motion blur, line features show higher robustness to represent the structural features of the environment and provide intuitive visual information (Zhao et al., 2022). By integrating object detection techniques with point-line fusion methods, the number of extractable point and line features can be ensured to be sufficient and well-distributed, thereby enhancing the stability of the system.

The main work of this paper is as follows:

1. Based on the ORB-SLAM2 algorithm, the target detection thread and dynamic region feature rejection module were added, while line feature extraction and point-line feature fusion were added to optimize the pose, and multi-thread parallelism was used to ensure the real-time capability and accuracy of the algorithm.
2. A target detection dataset based on inspection environment has been constructed for training target detection models. A lightweight target detection model named GNV2 was developed based on YOLOv8s, which was lightly processed by combining GhostNetV2. The CBAM (Convolutional Block Attention Module) attention mechanism and SCDown downsampling module have been added.
3. Integrated the GNV2 model into the improved SLAM algorithm and evaluate it, and deploy the GNV2-SLAM to the inspection robot for experiments.

The structured of this paper is organized as follows: Section II reviews the relevant literature in this research field and summarizes the current research progress. Section III elaborates the overall architecture of the GNV2-SLAM system and the improvement methodology. Section IV describes the experimental materials and research methodology used in this research. Section V presents the experimental results and evaluates the system’s performance based on the TUM dataset. Finally, Section VI concludes the major findings of this research. Section VII discusses contributions of this research work, and outlines potential directions for future research.

## 2 Related work

The feature point method is a widely utilized approach for visual mileage computation method in VSLAM. This method primarily focuses on extracting and matching key feature points across consecutive image frames to estimate the camera’s motion trajectory (Chen et al., 2018). To enhance the stability and robustness of SLAM systems in dynamic environments, deep learning techniques have been increasingly integrated in recent years to identify and eliminate dynamic feature points. Numerous researches have been devoted to integrating target detection and image segmentation methods from deep learning into SLAM systems. These advancements provide valuable *a priori* information for the recognition and eliminate dynamic feature points, thereby improving the performance of the system in complex scenarios (Favorskaya, 2023).

In order to achieve high-precision localization and map construction, Bescos et al. (2018) proposed Dyna-SLAM, which significantly improves the localization accuracy by identifying and eliminating the keypoints in the dynamic region through the Mask R-CNN (He et al., 2018) method. However, its real-time performance is poor due to its dependence on deep semantic segmentation. Yu et al. (2018) proposed DS-SLAM, which combines semantic segmentation with motion consistency detection to construct semantic maps and improve accuracy. Although these methods improve the accuracy, they generally suffer from insufficient real-time performance. Islam Q. U. et al. (2023) proposed YoloV8-SLAM, which employs the cutting-edge target detection algorithm YoloV8 and enhanced multiview geometry techniques to handle low, medium, and high-dynamic environments, whereas a well-matched point selection algorithm extracts high-speed motion information. Wu et al. (2022) proposed the YOLO- SLAM algorithm to accelerate and generate the basic semantic information of the SLAM system by Darknet19-YOLOv3 lightweight target detection network, and utilize the depth difference of random sample consistency to distinguish dynamic features. These algorithms can ensure the real-time operation of the SLAM system, but when there are multiple moving objects or image blurring in the environment, it will reduce the number of extracted feature points and cause the system to experience tracking failure.

Li et al. (2021) proposed RPL-SLAM by extracting point features and line features. The depth information of the RGBD image is further utilized to recover the 3D information of the point and line features, which improves the accuracy of the camera trajectory localization and solves the problem of not being able to find enough reliable features in case of missing texture or motion blur. Zhang, (2021) proposed PL-GM, which calculates the camera position by utilizing the two kinds of features of the point and line features, and constructs a 3D point element and line element by taking into account the two-dimensional point elements and line elements to constrain the error and enhance the positioning accuracy calculated by the algorithm. Although these algorithms improve the positioning accuracy of the system, these algorithms are only applicable to static environments, and when a dynamic target appears in the environment, it will lead to mis-correlation of data, which will cause the system to crash. Yuan et al. (2023). proposed PLDS-SLAM, a point and line fusion SLAM system for dynamic environments, which combines the *a priori* dynamic region detection, the geometrical and epipolar constraints to separate static and dynamic targets, and the introduction of Bayesian-based SLAM system with a point and line fusion. Wang et al. (2018) proposed a SLAM method that combines point and line features with real-time target detection to enhance the localization accuracy and robustness of the system by enhancing the feature extraction capability in an indoor environment and eliminating the interference of dynamic targets.

Cowsheds, as a typical structured scene, contain a large number of linear structures. However, the frequent appearance of dynamic targets often interferes with traditional SLAM systems during the feature extraction stage, leading to positioning errors. Additionally, when there are too many dynamic targets or motion blur, the number of effective feature points in the image decreases, negatively impacting the system’s positioning accuracy and operational reliability. To address these issues, this paper proposes a visual SLAM method based on dynamic target removal and point-line feature fusion, effectively enhancing the system’s accuracy and stability in real-world inspection scenarios.

## 3 Methodology

In this section, the GNV2-SLAM system is presented in detail. This system integrates a lightweight deep learning model optimization strategy, enabling it to achieve efficient target recognition and dynamic feature point elimination effectively. Furthermore, the SLAM system incorporates the point-line feature fusion strategy, which establishes foundation for the accurate localization and autonomous inspection of the mobile robot in cowshed inspection environment.

### 3.1 Overview of the GNV2-SLAM system

**FIGURE 1:**
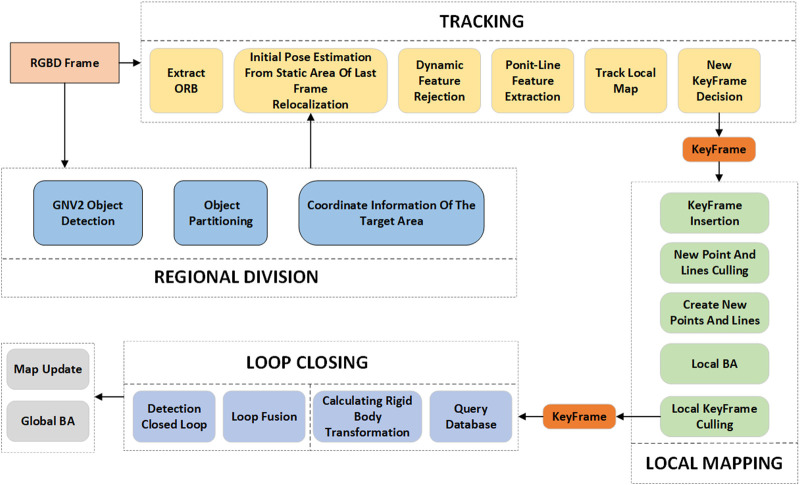
GNV2-SLAM system framework.

The framework of the GNV2-SLAM system proposed in this paper is shown in Figure 1. The system has been structurally optimized and functionally extended based on ORB-SLAM2 (Mur-Artal and Tardós, 2017). In addition to the original three threads: tracking, local map building, and closed-loop detection, a new target detection thread has been introduced to facilitate real-time recognition of dynamic targets. Concurrently, a dynamic target rejection module has been implemented to effectively remove the interference feature points caused by moving objects and improve the robustness of the system in dynamic environments. Furthermore, the system also performs line feature extraction and the optimizes of point-line feature fusion.

### 3.2 Overview of the GNV2 lightweight target detection network

**FIGURE 2:**
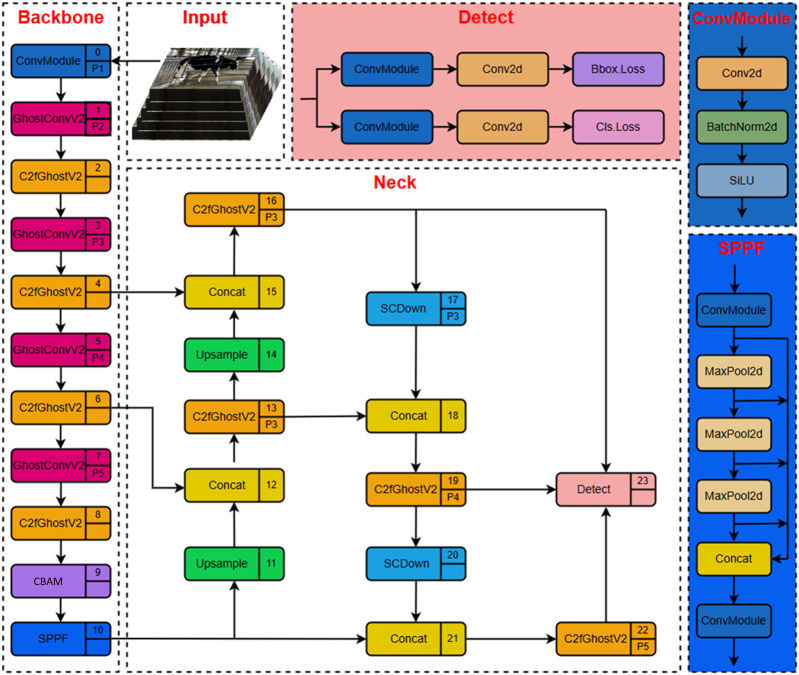
GNV2 network architecture.

The GNV2 network is based on YOLOv8s. An efficient GNV2 target detection network is constructed through a lightweight design and two structural optimizations, aiming to significantly reduce the consumption of computational resources and maintain high operational speed while ensuring detection accuracy. In this study, YOLOv8s′ original backbone network is substituted with lightweight GhostNetV2 to reduce model parameters and computation costs. To enhance the model performance further, two important improvements have been made to its network structure. First, the CBAM attention mechanism is introduced, which guides the model to pay more attention to the key fields related to the target by modeling the importance of the channel dimension and the spatial dimension, thus effectively improving the detection accuracy. Second, the SCDown downsampling module is adopted to optimize the feature downsampling process, so that the model achieves more efficient feature compression and delivery while maintaining the key feature information, thus enhancing the expressive capability of the overall network. The final structure of the GNV2 network is shown in Figure 2, which combines high accuracy and high efficiency and provides a good foundation for subsequent deployment in SLAM systems.

#### 3.2.1 GhostNetV2 neural network

**FIGURE 3:**
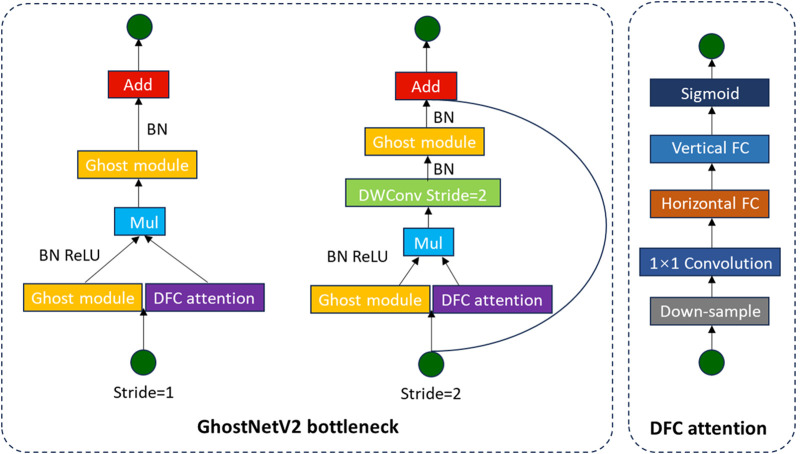
GhostNetV2 architecture schematic diagram.

GhostNetV2 (Tang et al., 2022) represents a lightweight convolutional neural network improved on GhostNet (Han et al., 2020), with the objective of improving feature expression capability while simultaneously reducing computational complexity. The core innovation lies in generating fundamental features using a limited number of convolutions via the Ghost module, subsequently producing additional redundant features through cost-effective linear operations. This approach effectively replaces traditional convolutional operations and significantly mitigates computational costs. In terms of structural design, the downsampling module of GhostNetV2 adopts stepwise convolution and pooling operations to minimize reliance on high-complexity operators, thereby further enhancing network efficiency. To enhance the feature representation capability of the intermediate layer, the network introduces the DFC (Dynamic Feature Consolidation) attention mechanism, which dynamically adjusts feature responses to enhance the expression of key features. Overall, GhostNetV2 effectively improves the balance between model accuracy and inference speed while maintaining a lightweight architecture. The basic module structure is shown in Figure 3.

#### 3.2.2 CBAM attention mechanism

**FIGURE 4:**
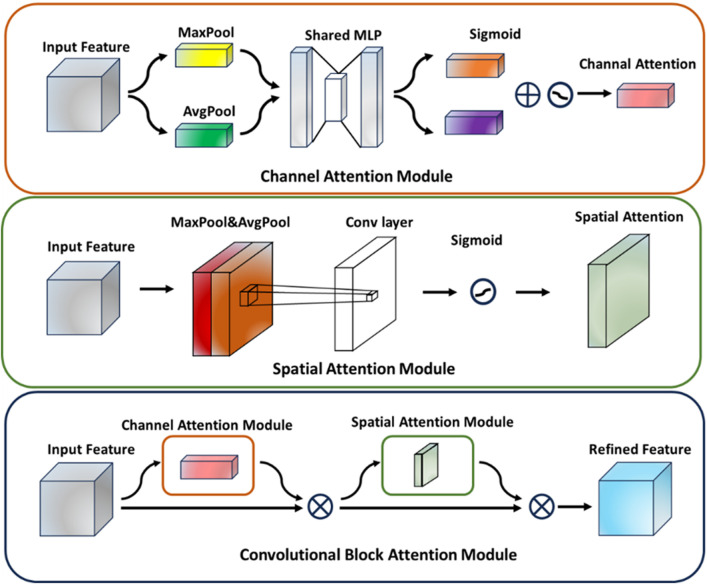
Cbam attention mechanism module structure.

Woo et al. (2018) proposed CBAM, a lightweight attention mechanism module designed to enhance the feature representation of convolutional neural networks. By sequentially integrating both Channel Attention and Spatial Attention mechanisms, this module directs the network to focus on key feature field more effectively, thus improving the performance of the model in various visual tasks. Specifically, CBAM first applies the channel attention module to the input feature map, extracts channel descriptive information through global maximum pooling and average pooling operations. It generates channel weight coefficients by combining these descriptors with a multilayer perceptron and weights the feature map along the channel dimension. Subsequently, this weighted feature map is passed into the spatial attention module, which extracts the spatial information through the pooling operations along the channel dimension and generates the spatial attention map by using convolution attention map. The feature map is weighted again in the spatial dimension. The final output feature map has stronger discriminative ability and can be used in the subsequent network structure to improve the overall performance. The structure of the CBAM attention mechanism module is shown in Figure 4.

#### 3.2.3 SCDown downsampling module

**FIGURE 5:**
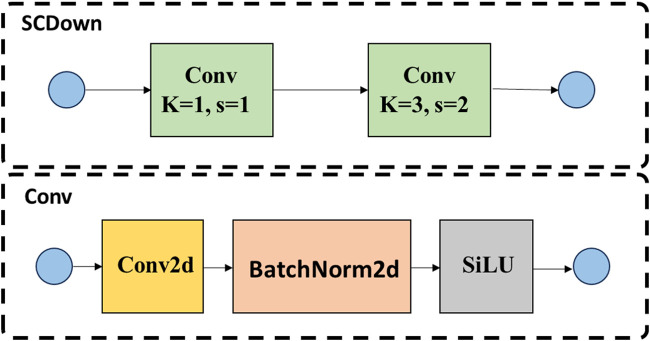
SCDown downsampling module architecture.

SCDown is an advanced downsampling module that simultaneously considers both spatial and channel dimensions. It is extensively utilized in lightweight neural networks and efficient computational models, with the objective of substantially decreasing computational complexity and parameter size while preserving feature integrity. By downsampling both the spatial dimensions and the number of channels of the input feature maps, this module decreases both the feature map size and the number of channels, thus effectively reducing the computational overhead and memory usage. In terms of implementation, the SCDown module usually combines convolutional operations, pooling layers or other downsampling strategies to retain key information while eliminating redundant features to improve the efficiency of feature processing. Its structural design provides an efficient feature compression scheme for lightweight networks, which helps to realize fast inference and deployment in resource-constrained environments. The structure of the SCDown module is shown in Figure 5.

### 3.3 Point and line feature fusion

In this paper, a line feature extraction and matching module is presented based on the ORB-SLAM2 framework. This enhancement enables the system to simultaneously extract point features and line features, so as to improve the robustness and stability of the VSLAM algorithm in complex scenarios. This improvement effectively enhances the system’s ability to perceive geometric information in structured environments by combining different types of feature information. Line features are extracted based on the LSD (Line Segment Descriptor) algorithm (Grompone Von Gioi et al., 2012), which extracts geometrically structured line segment features from images. To enhance the effectiveness of features and matching accuracy, line features are filtered and optimized based on the length of the line segments. Subsequently, LBD (Line Band Descriptor) algorithm descriptors are used to characterize the filtered line segments and match them with line features in other keyframes, so as to construct a stable line feature association relationship. By introducing the line feature information, the localization accuracy of the system is improved.

#### 3.3.1 Point feature reprojection error

**FIGURE 6:**
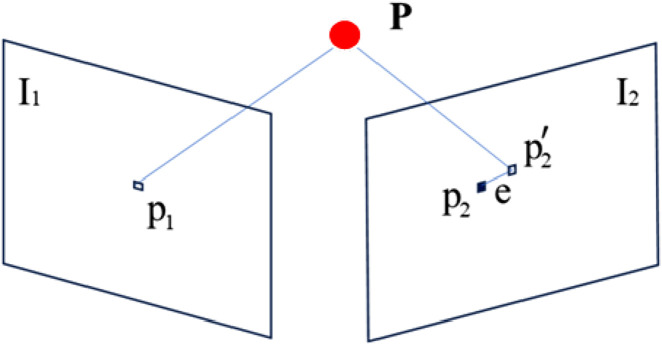
Reprojection error of point features.

The reprojection error is used to optimize the robot’s position. The reprojection error for the line features is shown in Figure 6. Let *I*_1_ and *I*_2_ denote two frames of images, the 3D spatial points *P* in the images corresponding to the pixel points *p*_1_ and *p*_2_, *p*_2_ *’* is the reprojection point of *p*_1_ on *I*_2_, and *e* is the error between *p*_2_ and *p*_2_ *´*.

The pixel coordinate of point *p*_2_ on *I*_2_ is *x*, *K* is the camera parameter matrix, *T*_*cw*_ is the bitmap transformation from the world coordinate system to the camera coordinate system between *I*_1_ and *I*_2_, and *X*_*w*_ is the coordinate of point *P* under the projection of *p*_1_ to the world coordinate system. *k* is the *k*th image with a total of *i* feature points. The reprojection error formula for feature points is shown in Equation 1.

$$e_{p,i}^{k}=x_{i}^{k}-I\left(KT_{cw}^{k}X_{w,i}^{k}\right)$$ (1)

#### 3.3.2 Line feature reprojection error

**FIGURE 7:**
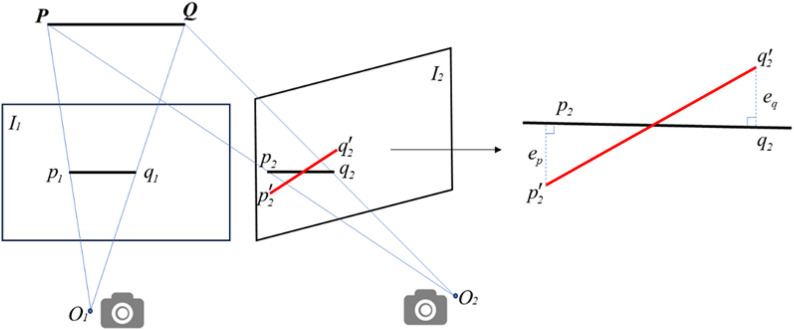
Reprojection error for line features.

The line feature reprojection error is shown in Figure 7. The *O*_1_ and *O*_2_ are the camera optical centers of the images, *I*_1_ and *I*_2_ are the two frames, *p*_1_ *q*_1_ and *p*_2_ *q*_2_ are the corresponding line features of the images, *p*_2_ *´q*_2_ *’* is the reprojected line segment of *p*_1_ *q*_1_ on *I*_2_, *e*_*p*_ is the error between *p*_2_ *’* and *p*_2_.

Plücker coordinates are commonly used to represent spatial line features, and the spatial line segment *PQ* expressed in Plücker coordinates as shown in Equation 2.

$$L_{w}=\left[\begin{array}{l} P\times Q\\ w_{1}P-w_{2}Q \end{array}\right]=\left[\begin{array}{l} n\\ v \end{array}\right]$$ (2)

The formula for projecting a line segment in the camera coordinate system to the pixel coordinate system is shown in Equation 3:

$$l_{c}=\left[\begin{array}{ccc} f_{x} & 0 & 0\\ 0 & f_{y} & 0\\ -f_{x}c_{x} & f_{x}c_{x} & f_{x}f_{y} \end{array}\right]n_{c}=Kn_{c}$$ (3)

The line segment in pixel coordinate system is denoted by *l*_*c*_, *f*_*x*_ and *f*_*y*_ represent the effective focal lengths along the x and y directions, respectively; *c*_*x*_ and *c*_*y*_ represent the translation of the origin of the coordinate system in the x and y directions, and the reprojection error of the line segment is shown in Equation 4.

$$e_{l,j}^{k}=d\left(l_{j}^{k},KT_{cw}^{k}L_{w,i}^{k}\right)$$ (4)

In the formula for calculating the reprojection error, the function *d* represents the orthogonal distance function from a point to a line.

Assuming that the observation errors of the point and line features are all Gaussian distributed cases, the combined error function *C* based on the point and line features can be obtained. As illustrated in Equation 5.

$$C=\sum_{k,i}\rho_{p}\left({e_{p,i}^{k}}^{T}\sum x_{k,i}^{-1}e_{p,i}^{k}+{e_{l,j}^{k}}^{T}\sum l_{k,j}^{-1}e_{l,j}^{k}\right)$$ (5)

$\sum l_{k,j}^{-1}$ is the covariance matrices of the line features, and $\sum x_{k,i}^{-1}$ is the covariance matrices of the point features. Respectively, and the Huber robustness cost function *ρ* is introduced to suppress the effect of outliers.

## 4 Equipment and methods

During the experiments, we ran the training task of the GNV2 deep learning model and the testing and evaluation work of the SLAM algorithm on the same server, respectively. Table 1 provides a detailed listing of the experimental environments used, including hardware configuration and software environment parameters.

**TABLE 1:** Experimental environment configuration.

Configuration	Equipment	Information
Hardware	CPU	Intel Core i7-12700
	GPU	NVIDIA GeForce RTX 2060
	RAM	16 GB
Software	System	Ubuntu 18.04
	Python	Python 3.9.19
Environment	Pytorch	1.12.1
	CUDA	11.3.1
	CuDNN	8.2.4

### 4.1 GNV2 model training

The YOLO image dataset utilized in this study comprises images captured using an Intel D455 camera. The dataset was collected from two large-scale dairy farms (Henan Ruiya Dairy Co., Ltd. and Luoyang Shengsheng Dairy Co., Ltd.) and the publicly available COCO dataset. We carefully selected a total of 1,246 images from these sources. These images were annotated with “Person” and “Cow” labels using the LabelImg tool. To improve the model’s generalization ability, data augmentation methods such as translation, mirroring, cropping, adding Gaussian noise, and adjusting brightness were used for offline expansion, ultimately constructing an enhanced dataset containing 6,230 images.

During model training, we used the Mosaic data augmentation method, which randomly selects four images and scales, rotates, crops, and rearranges them to generate new images for the model to learn. This method improves the model’s adaptability in multi-scale object detection through random combinations of multi-scale objects, thereby enhancing the diversity of the dataset. This proprietary dataset had strong generalizability and was suitable for train and evaluate a wide range of network models. The training and validation set were divided in a ratio of 8:2, while the test set consisted of video streaming images obtained during the actual operation of the GNV2-SLAM system. The hyperparameter configurations utilized for the training of the GNV2 network is shown in Table 2.

**TABLE 2:** Hyperparameter configuration for GNV2 network training.

Hyperparameter	Value	Hyperparameter	Value
Epoch	200	Weight_decay	0.0005
Batch Size	16	Mosaic Augmentation	1.0
Initial Learning Rate	0.01	Classification Loss Weight	0.5
Final Learning Rate Factor	0.01	Objectness Loss Weight	1.0

### 4.2 Performance evaluation of VSLAM algorithms

#### 4.2.1 TUM dataset

A publicly available TUM RGB-D dataset was used to evaluate the proposed system. The dataset, published by the Technical University of Munich, was acquired using a Kinect camera and contains time-synchronized color images, depth images, and camera positional truth (Ground Truth) from a high-precision motion capture system (Motion Capture). The image resolution is 640 × 480, which is suitable for evaluating the localization accuracy and robustness of various RGB-D SLAM algorithms in indoor environments (Singh et al., 2024). The TUM dataset encompasses a diverse range of scenarios, including low-texture environments, fast camera motion, illumination variations, and so on. These characteristics provide a rich set of samples for testing algorithms’ robustness and generalization capabilities. In this study, the representative sequences were selected such as fr3_sitting_halfsphere, fr3_walking_rpy, fr3_walking_xyz, among others. These sequences cover complex scenarios that ranging from low-dynamic environments to high-dynamic environments.

#### 4.2.2 Assessment methods

In order to quantitatively evaluate the trajectory estimation performance of the SLAM system, this study employed the evaluation tool provided by the TUM dataset to compare the camera trajectories outputted by our system against the true value trajectories included in the dataset. Absolute Trajectory Error (ATE) serves as one of the key metrics for evaluating the performance of SLAM system performance, which is used to quantify the positional deviation between the estimated trajectory of the system and the true trajectory (Ground Truth). ATE measures the spatial global consistency across entire trajectory while reflecting cumulative error over extended periods, thus providing an effective method to both algorithm robustness and localization accuracy. In addition to ATE analysis, this paper also incorporated Relative Pose Error (RPE) as a supplementary evaluation metrics. RPE focuses on the relative transformation error between neighboring frames, and can effectively evaluate the local accuracy and trajectory smoothness of the system in short time scales. This metric is especially suitable for analyzing the drift phenomenon caused by the instability of position estimation. By comparing the performance of different algorithms in the ATE and RPE dimensions, each algorithm’s actual performance in different scenarios can be more comprehensively revealed.

The formula used to calculate the absolute trajectory error between the estimated trajectory *Q* and the true trajectory *P* is shown in Equation 6.

$$ATE=\sqrt{\frac{1}{n}{\sum_{i=1}^{n}\left\|\log{\left(Q_{i}^{-1}P_{i}\right)}^{\vee }\right\|}_{2}^{2}}$$ (6)

Where, *n* is the number of trajectory points on the trajectory.

The relative trajectory error is shown in Equation 7.

$$RPE=\sqrt{\frac{1}{m}\sum_{i=1}^{m}\left\|\log{\left(Q_{i}^{-1}Q_{i+\Delta }\right)}^{-1}\left(P_{i}^{-1}P_{i+\Delta }\right)\right\||{|}_{2}^{2}}\begin{array}{cc} , & m=n-\Delta \end{array}$$ (7)

where, Δ is the time interval between two consecutive poses.

## 5 Experimental results

### 5.1 Results of the GNV2 experiment

In this study, significant emphasis has been placed on optimizing the structural complexity of the GNV2 model to minimize the computational resource consumption during the inference phase, while maintaining both accuracy and real-time performance in model detection. To comprehensively evaluate the model performance, metrics were utilized as follows: the target detection accuracy as measured by the average precision with an IoU threshold of 0.5 (mAP@0.5), the number of model parameters (Parameters), computational overhead measured by hundreds of billions of floating-point operations per second (GFLOPs), and the size of the model weights file are used to characterize the structural complexity of the model. The last three metrics collectively reflect the computational resource requirements of the model during deployment.

#### 5.1.1 Lightweight network comparison experiment

Three mainstream obtained lightweight feature extraction networks were replaced with the backbone network of the original YOLOv8s to obtain three lightweight variant networks. These lightweight variant networks would be trained on the self-constructed cowshed inspection environment dataset to generate the corresponding target detection models. The experimental results for the different lightweight variant networks are shown in Table 3.

**TABLE 3:** Comparative experiments on lightweight variant networks.

Network model	mAP@0.5/%	Params	GFLOPs	Weight/M
YOLOv8s (CSPLayer_2Conv)	94.15	11136374	28.6	22.5
YOLOv8s-VanillaNet	91.47	6892822	18.1	14.2
YOLOv8s-EfficientNet	91.17	6522578	17.3	13.4
YOLOv8s-GhostNetV2 (GNV2*)	94.07	6983726	18.5	14.4

The experimental results indicated that replacing the original backbone network of YOLOv8s with VanillaNet (Chen et al., 2023), EfficientNet (Tan and Le, 2020) and GhostNetV2, leads to a significant reduction in the number of parameters, computational load, and the model size. Although there is a decrease in detection accuracy, the extent of this reduction varies among different models. Specifically, YOLOv8s-VanillaNet exhibits a decline in detection accuracy by 2.68%, resulting in an accuracy of 91.47%. Conversely, YOLOv8s-EfficientNet experiences the most substantial decrease in accuracy at 2.98% while achieving a remarkable reduction in computational workload by 39.51%. In contrast, YOLOv8s-GhostNetV2 (GNV2*) mitigates model complexity while preserving detection accuracy, it showed only a minor drop of 0.08% in average detection accuracy along with notable reductions: 37.29% fewer parameters, 35.31% decrease in computation load, and 36% reduction in the model size. The comprehensive performance advantages were so evident that GNV2* was selected as the foundational network for further enhancements within this study.

#### 5.1.2 Comparative experiments on attention mechanisms

Three different attentional modules, such as EffectiveSE, MHSA and ECA, were selected for comparison experiments with CBAM channel attentional modules. The results of the comparison experiments of the attention mechanisms are shown in Table 4.

**TABLE 4:** Attention comparison experiment.

Network model	mAP@0.5/%	Params	GFLOPs	Weight/M
GNV2*	94.07	6983726	18.5	14.4
GNV2*-CBAM	94.48	6494864	18.2	13.4
GNV2*-EffectiveSE	94.26	6735070	18.5	14.3
GNV2*-MHSA	92.18	7249966	18.9	14.9
GNV2*-ECA	94.43	6472417	18.5	13.2

The experimental data indicate that the introduction of the MHSA attention mechanism not only increases the model’s complexity but also reduces its accuracy. After increasing the EffectiveSE attention mechanism, there was a slight enhancement in average detection accuracy compared to the GNV2* model, while the number of parameters and the model size were also reduced. A comparative analysis between the ECA and CBAM attention mechanism revealed minimal differences regarding their respective advantages. Although the ECA attention mechanism offers the advantage of a smaller model, the introduction of the CBAM attention mechanism resulted in the highest average detection accuracy, improving by 0.41% compared to the original GNV2* model, while also achieving the lowest floating-point computation. Therefore, the incorporation of the CBAM attention mechanism significantly enhances the performance of the GNV2* model. Consequently, the introduction of CBAM attention mechanism more effectively enhances the performance of the GNV2* model.

#### 5.1.3 Downsampling module comparison experiments

Through the comparison experiments of different attention mechanisms, the results showed that introducing the CBAM attention mechanism into the GNV2* model significantly improves its accuracy. On the basis of this finding, the superiority of the model performance after the introduction of the SCDown downsampling module was verified by comparing with the ADown and RFCAConv downsampling modules. The results from these downsampling comparison experiments are shown in Table 5.

**TABLE 5:** Downsampling comparison experiments.

Network model	mAP@0.5/%	Params	GFLOPs	Weight/M
GNV2*-CBAM	94.47	6494864	18.2	13.4
GNV2*-CBAM-SCDown	95.19	6464998	18.1	13.4
GNV2*-CBAM-ADown	94.19	6451246	17.8	13.3
GNV2*-CBAM-RFCAConv	94.68	7031774	18.6	14.5

The experimental data indicate that the introduction of the ADown downsampling operator decreases the floating-point computations, the number of parameters and the model size significantly compared with the initial model GNV2*-CBAM model. However, this improvement is accompanied by a slight decrease in the average accuracy of 0.28%. The introduction of the RFCAConv downsampling operator enhances the model complexity while increasing the detection accuracy. The model accuracy improved the most when the SCDown operator was combined with the original model, reaching 95.19%, while the average model accuracy improved by 0.75%. In addition, the number of parameters and floating-point calculations were also low, enabling a better balance between efficiency and performance. This enables a more favorable balance between efficiency and performance. Thus, the final model obtained by fusing GNV2* lightweight network, CBAM attention mechanism and SCDown downsampling was designated as GNV2.

#### 5.1.4 Algorithm comparison experiments

The GNV2 model was compared with different models of YOLO series to highlight the performance advantages of the algorithms proposed in this study. The data of average detection accuracy and model size were used to compare the performance difference of different algorithms on the self-constructed cowshed inspection dataset. The results of comparison experiments of different algorithms of YOLO series are shown in Table 6.

**TABLE 6:** Comparison experiment of different algorithms of YOLO series.

Network model	mAP@0.5/%	Weight/M
YOLOv5n	88.81	5.3
YOLOv5s	93.14	18.5
YOLOv8n	91.26	6.3
YOLOv8s	94.15	22.5
YOLOv10n	89.65	5.8
YOLOv10s	92.9	16.5
GNV2	95.19	13.4

**FIGURE 8:**
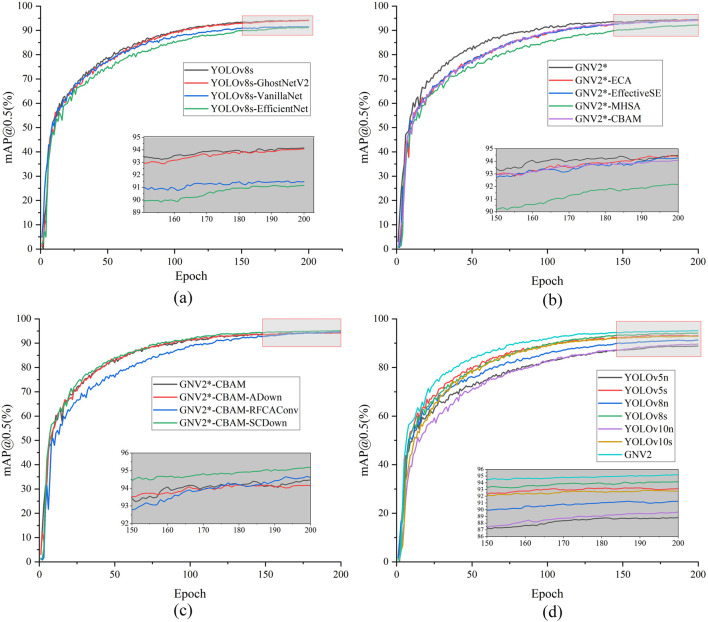
mAP@0.5 graph. **(a)** The mAP@0.5 curves of different models in the lightweight network comparison experiment; **(b)** The mAP@0.5 curves of different models in the attention comparison; **(c)** The mAP@0.5 curves of different models in the downsampling comparison experiment; **(d)** The mAP@0.5 curves of different models in the algorithm comparison experiment.

The experimental results indicate that GNV2 surpasses other models within the YOLO series in terms of average detection accuracy. It achieved an accuracy that was still 3.93% higher than that of the highest-accuracy YOLOv8n, even though it does not have the advantage of YOLOv5n, YOLOv8n, and YOLOv10n in terms of model size. In comparison to its predecessor YOLOv8s, the GNV2 model size was reduced by 40.44%, rendering it 3.1M smaller than YOLOv10s while also improving its accuracy by 2.29%. In conclusion, GNV2 effectively balances high accuracy with substantial model compression requirements while considering both performance metrics and lightweight design. The mAP@0.5 performance curves of each model in different experiments are shown in Figure 8.

**FIGURE 9:**
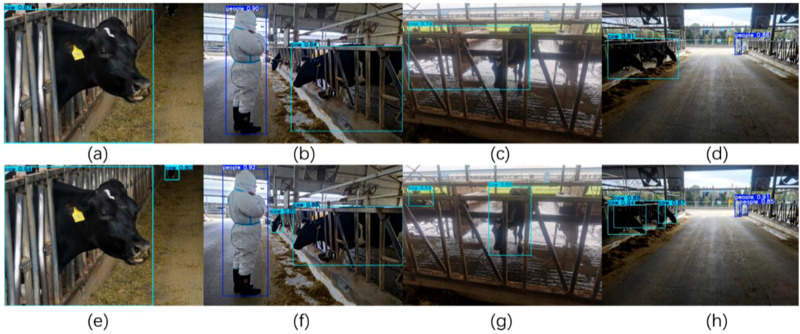
Comparison of the detection results of YOLOv8s and GNV2. The images in the first row **(a–d)** represent the detection results of YOLOv8s in four images; the images in the second row **(e–h)** represent the detection results of GNV2 in the same four images as YOLOv8s.

The results presented in Figure 8 provide validation for the effectiveness of the selected strategies at each stage. Furthermore, a comparative analysis was conducted between the detection accuracy of the GNV2 model and that of the YOLOv8s to evaluate its improvement. The detection results are shown in Figure 9, demonstrating that the overall detection accuracy of GNV2 surpasses that of YOLOv8s network.

### 5.2 GNV2-SLAM experimental results

The GNV2 model was integrated into the GNV2-SLAM system for performing the target recognition task. Subsequently, the overall performance of GNV2-SLAM as well as the tracking elapsed time were evaluated on the TUM dataset. Using the ORB-SLAM2 as a reference, the performance difference between GNV2-SLAM and Dyna-SLAM were further compare. All algorithms were executed independently for five times under identical conditions, and the final results were averaged as the evaluation metrics.

#### 5.2.1 Effectiveness of dynamic feature removal and point and line feature fusion

**FIGURE 10:**
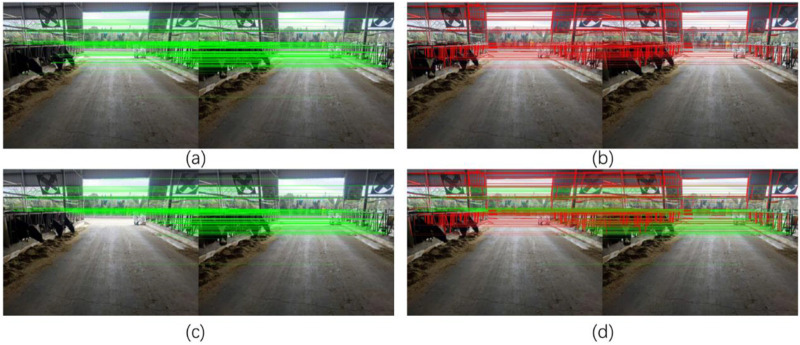
Different feature extraction results **(a)** shows the effect of extracting only ORB feature points and matching, some of the feature points were located on the cow, which may affect the subsequent calculation of the SLAM system and cannot guarantee the accuracy of localization, and Figure **(b)** shows the effect of matching after eliminating the feature points on the dynamic target. Figure **(c)** shows the effect of extracting LSD line features and matching. Figure **(d)** shows the effect of removing the dynamic region as well as point-line fusion proposed in this paper.

In order to verify the effectiveness of the point and line feature extraction algorithm, experiments were conducted on different images in the cowshed inspection dataset. The images following feature extraction and matching are shown in Figure 10.

#### 5.2.2 TUM dataset for performance evaluation

The performance of the SLAM algorithm was evaluated by selecting the root mean square error (RMSE) and standard deviation (SD) in low-dynamic environments (fr3_s_hs) and high-dynamic environments (fr3_w_hs, fr3_w_rpy, etc.). The results pertaining to absolute trajectory error, comparison of relative positional error translational drift, comparison of relative positional error rotational drift for GNV2-SLAM algorithm, ORB-SLAM2 algorithm, and Dyna-SLAM algorithm are shown in the tables as Tables 7-9 respectively.

**TABLE 7:** Absolute trajectory error results for different algorithms.

Sequences	ORB-SLAM2		Dyna-SLAM		GNV2-SLAM		Improvement against ORB-SLAM2	
	RMSE	SD	RMSE	SD	RMSE	SD	RMSE (%)	SD (%)
fr3_s_hs	0.066	0.0355	0.0287	0.0142	0.0271	0.0133	58.94	62.54
fr3_w_hs	0.5082	0.2341	0.0271	0.0133	0.0273	0.0138	94.63	94.11
fr3_w_rpy	0.7604	0.3739	0.0442	0.0214	0.0446	0.0283	94.13	92.43
fr3_w_static	0.0596	0.0341	0.0102	0.0047	0.0099	0.0038	83.39	88.86
fr3_w_xyz	0.6819	0.34	0.0325	0.0175	0.0264	0.0142	96.13	95.82

**TABLE 8:** Relative trajectory translation error results for different algorithms.

Sequences	ORB-SLAM2		Dyna-SLAM		GNV2-SLAM		Improvement against ORB-SLAM2	
	RMSE	SD	RMSE	SD	RMSE	SD	RMSE (%)	SD (%)
fr3_s_hs	0.0358	0.0239	0.0243	0.0137	0.0158	0.0093	55.87	61.09
fr3_w_hs	0.1633	0.1301	0.0236	0.0124	0.0227	0.0116	86.1	91.08
fr3_w_rpy	0.1765	0.1357	0.0358	0.0203	0.0486	0.0314	72.46	76.86
fr3_w_static	0.0477	0.0411	0.0096	0.0047	0.0089	0.0049	81.34	88.08
fr3_w_xyz	0.1701	0.1154	0.0207	0.0106	0.0198	0.0099	88.36	91.42

**TABLE 9:** Relative trajectory rotation error results for different algorithms.

Sequences	ORB-SLAM2		Dyna-SLAM		GNV2-SLAM		Improvement against ORB-SLAM2	
	RMSE	SD	RMSE	SD	RMSE	SD	RMSE (%)	SD (%)
fr3_s_hs	0.0161	0.0082	0.0155	0.0074	0.0131	0.0058	18.63	29.27
fr3_w_hs	0.0842	0.065	0.0157	0.0083	0.0153	0.0079	81.83	87.85
fr3_w_rpy	0.0867	0.066	0.0189	0.0107	0.0238	0.0149	72.55	77.42
fr3_w_static	0.0213	0.0174	0.0065	0.0027	0.0063	0.0029	70.42	83.33
fr3_w_xyz	0.0811	0.0539	0.0113	0.0057	0.0112	0.0056	86.19	89.61

**FIGURE 11:**
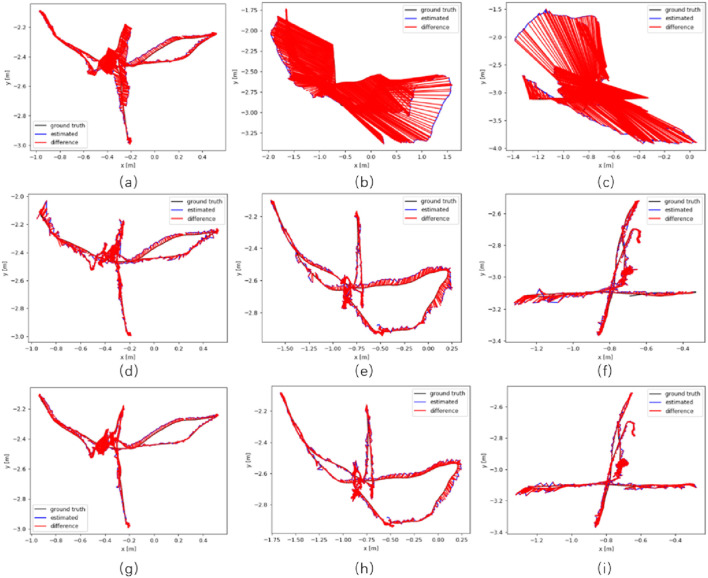
Absolute trajectory error maps Image **(a,d,g)** represents the ATE maps of ORB-SLAM2, Dyna-SLAM, and GNV2-SLAM on fr3_w_hs sequences, respectively; image **(b,e,h)** represents the ATE maps of the three algorithms on fr3_w_hs sequences; image **(c,f,i)** represents the ATE maps of the three algorithms on fr3_w_xyz sequence.

The experimental results indicate that GNV2-SLAM significantly outperforms ORB-SLAM2 in terms of absolute trajectory error in high-dynamic environments, with RMSE decreasing exceeding 83.39% and SD decreasing exceeding 88.86%. Compared with Dyna-SLAM, GNV2-SLAM exhibited superior localization accuracy in low-dynamic scenarios while showing comparable performance in high-dynamic scenarios. In contrast, the GNV2 network can effectively identify dynamic targets, improving the robustness of the system in dynamic environments. Regarding relative trajectory error, the relative position error of GNV2-SLAM was lower than that of ORB-SLAM2 in low dynamic scenarios. with slight improvements observed in accuracy. Conversely, in high dynamic scenarios, the translation error RMSE decreases by up to 88.36%, and the SD decreases by up to 91.42%. The trend of rotation error was also consistent. In some sequences, the overall performance of GNV2-SLAM was better than that of Dyna-SLAM, and Figure 11 shows the comparison of the absolute trajectory error (ATE) of ORB-SLAM2, Dyna-SLAM and GNV2-SLAM in some sequences. The results indicate that the error of GNV2-SLAM was significantly reduced and exhibits higher localization accuracy.

#### 5.2.3 Tracking time assessment

VSLAM systems needs to strike an optimal balance localization accuracy and real-time performance. To evaluate the real-time performance of these algorithms, this study compares the average tracking time of ORB-SLAM2, Dyna-SLAM and GNV2-SLAM using the TUM dataset. In this experiment, the average time for each algorithm to process a single image frame were counted, and measured the time consumption of the tracking process. The results were in seconds, and are shown in Table 10.

**TABLE 10:** Time consumption of the tracking process.

Sequences	ORB-SLAM2	Dyna-SLAM	GNV2-SLAM
fr3_s_hs	0.019	1.717	0.029
fr3_w_hs	0.02	1.827	0.023
fr3_w_rpy	0.02	1.779	0.027
fr3_w_static	0.018	1.765	0.024
fr3_w_xyz	0.021	1.764	0.028

The experimental data analysis revealed that although GNV2-SLAM has a slight increase in time overhead compared to ORB-SLAM2 after the introduction of the target detection threading processes, the system can still successfully complete single frame image tracking within approximately 29 ms, which provides a significant advantage in real-time performance. Compared with Dyna-SLAM, GNV2-SLAM demonstrates an approximate reduction in processing time by about 90%, further highlighting its substantial benefit in terms of real-time performance.

#### 5.2.4 Assessment in real environments

**FIGURE 12:**
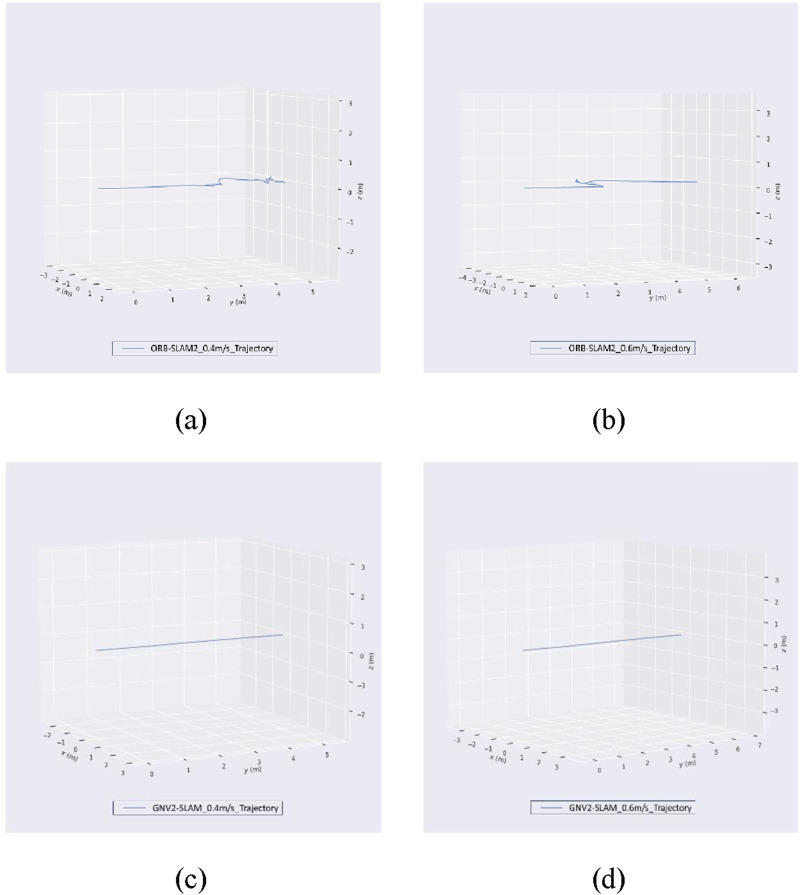
Absolute trajectory error maps Images **(a,b)** represent the trajectory maps computed by ORB-SLAM2 at 0.4 m/s and 0.6 m/s velocity on the robot, respectively. Image **(c,d)** represents the trajectory maps computed by GNV2-SLAM2 at 0.4 m/s and 0.6 m/s velocity on the robot, respectively.

In order to verify the effectiveness of the proposed algorithm, it was deployed the algorithm to a cowshed inspection robot and tested the localization accuracy of the GNV2-SLAM in a cowshed inspection environment. The experiments used an NVIDIA Jetson Xavier NX as the robot’s upper computer with Ubuntu 18.04 operating system and configured with a Melodic version of the ROS (Robot Operating System) system. The inspection robot moves along a straight line at speeds of 0.4 m/s and 0.6 m/s while employing the GNV2-SLAM system and the ORB-SLAM2 system, respectively. The resulting trajectories from different vision SLAM systems are shown in Figure 12.

From the experimental results, it could be seen that during the robot’s movement along a straight line, the interference of dynamic targets occurs, which leads to an obvious drift in the trajectory of ORB-SLAM2. GNV2-SLAM utilizes the line features in the cowshed environment to perform point and line fusion to make the trajectory closer to the real trajectory while eliminating the dynamic targets.

## 6 Discussion

This paper proposes an improved GNV2-SLAM method based on the ORB-SLAM approach. The method maintains high accuracy while significantly enhancing the system’s real-time performance. By refining the GNV2 model, the system’s robustness and accuracy in object recognition are improved. GNV2-SLAM demonstrates superior precision in high-dynamic environments, particularly excelling in object recognition and localization accuracy, which highlights its strong model compression capability and efficient extraction of point and line features.

It is worth noting that in certain high-dynamic environments, GNV2-SLAM still shows some gaps compared to Dyna-SLAM. The main reason lies in Dyna-SLAM’s ability to leverage prior dynamic region information from each frame to achieve pixel-level semantic segmentation, thus providing stronger dynamic point filtering capabilities that effectively enhance the precision of static environment mapping. The global optimization strategy of GNV2-SLAM may lead to instability or misoptimization in dynamic scenes due to frequent interference from dynamic objects. However, comparing the Relative Pose Error (RPE) results shows that GNV2-SLAM, through point-line feature fusion, significantly reduces computational errors, effectively optimizing the pose estimation process and improving the system’s accuracy and stability.

However, due to budget constraints, the research has primarily focused on the algorithm’s effectiveness, and hardware experiments still require improvement. Future work will focus on the adaptation of the algorithm to complex hardware platforms, aiming to achieve automated monitoring of livestock activity, spatial distribution, and facility safety. Additionally, multiple metrics will be used to comprehensively assess the adaptability and application potential of GNV2-SLAM in dynamic agricultural environments.

## 7 Conclusion

This paper presents a vision-based SLAM method, GNV2-SLAM, which integrates dynamic object removal and point-line feature fusion for livestock barn inspection. Building on ORB-SLAM2, the method introduces a target detection thread and a dynamic point removal module. By adopting a lightweight design for the target detection model, the overall computational complexity is reduced. Additionally, the CNAM attention mechanism and SCDown downsampling structure are incorporated to further optimize performance without increasing model complexity. This model is integrated into the SLAM system, and point and line features are fused to improve pose estimation.

Experimental results show that, compared to YOLOv8s, the GNV2 network model achieves a 1.04% improvement in average detection accuracy, with a 41.96% reduction in the number of parameters, a 36.71% decrease in computational load, and a 40.44% reduction in model size. After integrating GNV2-SLAM into the visual SLAM system, performance evaluation on the TUM dataset demonstrates that GNV2-SLAM outperforms ORB-SLAM2 in high-dynamic scenes, achieving a reduction of over 83.39% in RMSE and 88.86% in SD for absolute trajectory error. For relative trajectory error, the translation error RMSE shows a maximum reduction of 88.36%, with SD reduced by 91.42%, and the rotation error follows a similar trend to the translation error. In the tracking evaluation, GNV2-SLAM processes each frame of the image within 30 ms, highlighting its excellent real-time performance and competitive advantage. Real-world evaluation results show that the trajectory generated by the proposed algorithm more accurately reflects the robot’s motion path in the actual environment.

Given the challenges posed by strong ground reflections and frequent target occlusions in livestock barn environments, future research will consider introducing a multimodal perception mechanism. By integrating thermal imaging, LiDAR, and visual data, the fusion of multimodal data and a deep learning-optimized multi-input network structure can enhance the system’s robustness and adaptability, improving target recognition and localization accuracy, and overcoming the limitations of GNV2-SLAM in occlusion and reflection scenarios. Furthermore, considering the dynamic nature of livestock, future work will focus on further optimizing dynamic object modeling and segmentation strategies to enhance map consistency and localization accuracy under conditions of frequent animal movement.

## Data Availability

The raw data supporting the conclusions of this article will be made available by the authors, without undue reservation.
